# Serum microRNA profiling to distinguish papillary thyroid cancer from benign thyroid masses

**DOI:** 10.1186/s40463-015-0083-5

**Published:** 2015-09-05

**Authors:** M. Elise R. Graham, Robert D. Hart, Susan Douglas, Fawaz M. Makki, Devanand Pinto, Angela L. Butler, Martin Bullock, Matthew H. Rigby, Jonathan R. B. Trites, S. Mark Taylor, Rama Singh

**Affiliations:** Division of Otolaryngology, Queen Elizabeth II Health Sciences Centre and Dalhousie University, Halifax, NS Canada; NRC Human Health Therapeutics, Oxford Street, Halifax, NS Canada; Division of Anatomical Pathology, Queen Elizabeth II Health Sciences Centre and Dalhousie University, Halifax, NS Canada

**Keywords:** Papillary thyroid cancer, MicroRNA, Serum testing, Biomarkers

## Abstract

**Objectives:**

Papillary thyroid cancer (PTC) is increasing in incidence. Fine needle aspiration is the gold standard for diagnosis, but results can be indeterminate. Identifying tissue and serum biomarkers, like microRNA, is therefore desirable. We sought to identify miRNA that is differentially expressed in the serum of patients with PTC.

**Methods:**

Serum miRNA was quantified in 31 female thyroidectomy patients: 13 with benign disease and 18 with PTC. qPCR results were compared for significant fold-changes in 175 miRNAs, against a pooled control.

**Results:**

128 miRNA qualified for analysis. There were identifiable fold-changes in miRNA levels between benign and control, and between PTC and control. There were statistically significant fold changes in the level of four miRNAs between benign and PTC: hsa-miR-146a-5p and hsa-miR-199b-3p were down-regulated, while hsa-let7b-5p and hsa-miR-10a-5p were up-regulated.

**Conclusions:**

MicroRNA is differentially expressed in the serum of patients with PTC. Serum miRNA has the potential to aid in thyroid cancer diagnosis.

## Background

Papillary thyroid carcinoma (PTC) accounts for up to 90 % of thyroid carcinomas, yet distinguishing carcinoma from benign thyroid conditions such as follicular adenomas, cysts and goiter can be difficult [1]. Thirty percent of fine needle aspirate specimens from thyroid nodules are indeterminate, and up to 75 % of patients undergo a hemithyroidectomy for benign disease unnecessarily [2–5]. As the incidence of PTC increases, along with the detection of incidental thyroid nodules on CT and ultrasound, research into the development of more sophisticated means of diagnosis is needed.

Recent research on the development of such diagnostic tools has focused on determining thyroid carcinoma gene expression profiles to identify markers of benign or malignant pathology [6]. Such markers, traditionally tested in tumor tissue, have been used to stratify indeterminate fine needle aspiration results to those more likely to be PTC versus benign nodules. Examples of potential markers that appear to be consistently up-regulated in PTC tissue samples are chitinase 3 like-1 (YKL-40), galectin-3 (Gal-3), cytokeratin 19 (CK19), tissue inhibitor of metalloproteinases-1 (TIMP-1) and angiopoetin-1 (Ang −1) [7–10].

An alternative to tumor tissue sampling is serum-based diagnostic testing. Serum testing would potentially allow for minimally invasive, safe and repeatable diagnosis, requiring less technical skill for collection. Although the feasibility of potential blood-based diagnostic testing has been shown [11], no biomarkers have been identified that reliably reflect elevated tumor tissue levels.

MicroRNAs (miRNAs) are non-coding RNA molecules involved in the regulation of gene expression [12]. miRNAs bind to messenger RNA (mRNA) and promote their degradation or prevent their translation into proteins. Over 2000 miRNA have been identified in the human genome, and more are being identified at a rapid rate [13]. The first miRNA was incidentally identified in 1993; however, its function as a positive and negative bioregulator was not recognized until the early 2000s [14, 15]. Deregulation of miRNA has been associated with a number of diseases, including malignancies such as chronic lymphocytic leukemia, colon cancer, glioblastoma and astrocytoma [16–19]. Traditionally, miRNAs have been detected in tissue specimens; however, recent studies have also shown that miRNAs remains relatively stable and detectable in blood samples [20, 21].

Numerous independent studies have identified miRNA expression profiles in differentiated thyroid carcinoma tissue, suggesting they may play a role in the development and progression of this carcinoma. miRNA profiles identified in thyroid cancer have included miRNA-221, −222 and -181b [22, 23]. It is possible that these unique miRNA may allow for improved diagnosis, prognosis and monitoring response to treatment in thyroid carcinoma.

The objective of this study was to identify potential miRNA serum markers in patients with PTC. We hypothesized that, given the stability of miRNA in serum compared to traditional biomarkers, patients with PTC would show a measureable change in the select miRNA levels relative to patients with benign lesions.

## Materials and methods

### Patient sampling

This study was approved by the Capital Health Research Ethics Board and informed consent was obtained from all participants.

Blood samples (approximately 15 mL) were collected in EDTA tubes from patients at the time of intravenous catheter placement in the operating room, prior to the administration of any anesthetic. A dedicated research nurse prepared serum samples from whole blood by centrifugation to separate serum from other blood components. Serum was aliquoted into 1.5 mL Eppendorf tubes, which were de-identified, barcoded and stored at −80 °C for future use. This process generated a biobank of 205 samples. From these, 45 samples (26 PTC and 19 benign) were randomly selected from female patients with thyroid masses greater than 1 cm. Separate RNA extractions were performed on three aliquots of a commercially available serum samples from a pool of twenty healthy individuals (Precision Biologics, Dartmouth, NS, Canada) for use as controls. These samples were screened for the presence of hemolyzed red cells using the NanoDrop™ 1000 spectrophotometer (NanoDrop Products, Wilmington, DE, USA) to detect optical density at 415 nm. Samples with evidence of hemolysis were excluded prior to further analysis, leaving 31 samples (13 benign, 18 PTC) (Table 1).

**Table 1:** Sample selection

	# of samples	Female	Tumor >1 cm	Passed hemolysis
Benign	118	87	19	13
PTC	87	50	26	18
Control	3	-	-	3

Serum sample collection. Control samples came from a commercially available pooled serum sample of 20 healthy individuals

### RNA isolation

Total RNA was extracted from 200 μL serum using the miRNeasy® Mini Kit (Qiagen, Mississauga, ON, Canada) as described by the manufacturer. A mixture containing 1.25 μg/mL of MS2 bacteriophage RNA, and RNA spike-ins was added to the serum. RNA was purified on an RNeasy® Mini spin column (Qiagen), eluted in 50 μL of RNase-free water, and stored at −80 °C.

### Sample quality control using spike-ins

To ensure that the quality of the input RNA was sufficiently high for effective reverse transcription (RT), cDNA synthesis and amplification, two types of RNA spike-ins were used. RNA isolation controls (UniSp2 and UniSp4) were added to the sample prior to purification and used to detect differences in extraction efficiency and the presence of inhibitors. The cDNA synthesis control (UniSp6) was added in the RT reaction to evaluate cDNA synthesis.

### Reverse Transcription (RT) and RT-qPCR

For quality assessment, qPCR assays for miRNA-103, −191, −23a, −30c, and −451 were performed. These miRNAs are expressed at a predictable level in the majority of sample types and are used to evaluate baseline miRNA content in the samples. A second hemolysis test was performed at this time using two of these microRNA: miR-451, which is increased in hemolyzed red blood cells compared to miR-23a, which is relatively stable in serum and plasma. A difference in Ct values (miR-23a minus miR-451) <8.0 qualified samples for analysis. Negative controls excluding template from the RT reaction were performed and profiled with the samples. Total RNA (2 μL) was reverse transcribed in 10 μL reactions using the miRCURY LNA™ Universal RT microRNA PCR, Polyadenylation and cDNA synthesis kit (Exiqon, Woburn, MA, USA). Each RT was performed in duplicate, including an artificial RNA spike-in (UniSp6). cDNA was diluted 50-fold and assayed in 10 μL qPCR reactions according to the protocol for miRCURY LNA™ Universal RT microRNA PCR (Exiqon).

miRNAs were profiled using the microRNA Ready-to-Use PCR human serum/plasma panel containing 175 miRNAs plus controls (Exiqon). 5 μL RNA was reverse transcribed in 25 μL reactions and assayed as described above. Each microRNA was assayed once by qPCR and negative controls excluding template from the RT reaction were included. PCR was performed in a LightCycler® 480 Real-Time PCR System (Roche Applied Science, Laval, QC, Canada) in 384-well plates. The amplification curves were analyzed using the Roche LC software, both for determination of expression values (2nd derivative Ct method) and for melting curve analysis.

### Data analysis and statistics

The raw data was extracted from the LightCycler 480 software. For quality control samples, an average Ct was calculated for the duplicate RTs, and evaluation of expression levels was performed based on raw Ct-values. The amplification efficiency was calculated using algorithms similar to the LinReg software [24]. All assays were inspected for distinct melting curves to ensure that the Tm was within the known specifications for the assay.

Statistics (limma expression analysis, including t-tests and false-discovery rates) were done on the R platform using the HTqPCR package [25]. Features where values were missing in more than 50 % of samples from any group (PTC, benign, control) and features where the negative controls were within 5 Ct values of the experimental Ct values were removed from the analysis. Data for microRNAs that were present in all 31 samples were normalized using norm rank-invariant normalization because it yielded the lowest standard deviations in the control replicates. For differential expression analysis, features with standard deviation >0.5 in the control group were removed. Random forest analysis was performed in the R platform using the random Forest package [26] and the ROCR package [27].

Gene targets for differentially expressed microRNAs were identified using a microRNA target prediction and annotation database (mirDB) using a prediction score of ≥80 (www.mirDB.org).

## Results

The consistent levels of all assays using RNA spike-ins (Uni-Sp2, Uni-Sp4 and Uni-Sp6) show that the RNA extraction as well as RT efficiency was similar for all samples, and that both RT and qPCR were successful. The baseline level, which is derived from the no-template control RT reaction, also shows that none of the samples contain inhibitors. Quality assessment of all positive and negative controls using qPCR assays for the commonly expressed miR-103, −191, −23a, −30c, and −451 showed Ct values between 22–24 for the positive control and ~40 for the negative control, indicating that the RT reaction worked well. The expression levels for all of the tested samples for these miRNAs were comparable to each other and to what is seen for other samples of similar types. Furthermore, the difference in Ct between miR-23a and miR-451 are 8.0 or lower in all samples, indicating minimal signs of hemolysis. Based on these quality control data we proceeded with the miR-panel profiling of all passed samples.

**Fig. 1:**
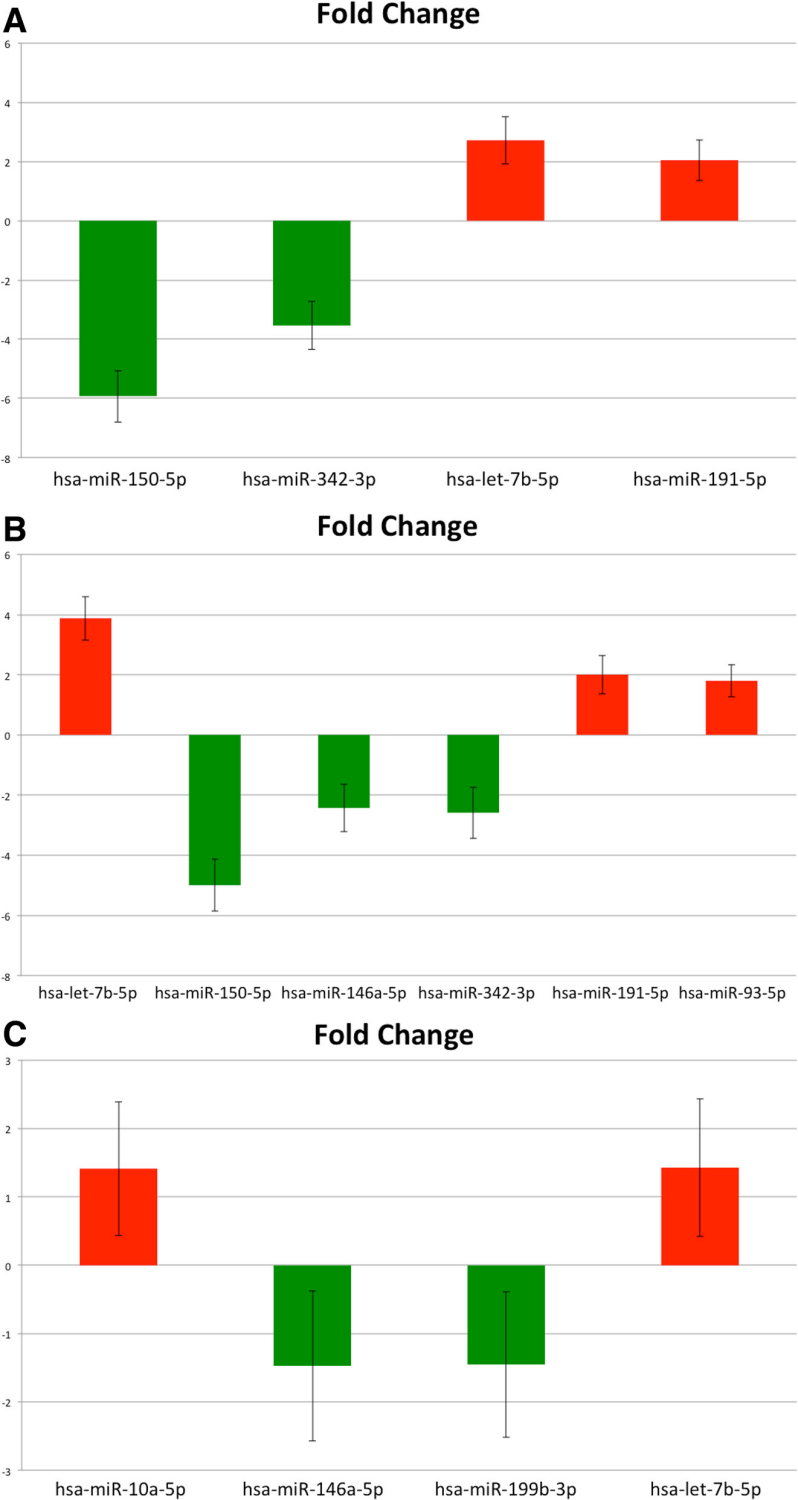
**a** Fold change in benign vs. control samples. Significant fold-changes were demonstrated in expression of four miRNAs between benign and control samples. **b** Fold change in PTC vs. control samples. Significant fold changes were demonstrated in expression of six miRNAs between PTC and control serum. Four of these were common to the benign vs. PTC comparison. **c** Fold change in PTC vs. benign samples. Statistically significant up-regulation of hsa-miR-10a-5p and hsa-let-7b-5p was seen between the serum of PTC vs. Benign patients, and statistically significant down-regulation of hsa-miR-146a-5p and hsa-miR-199b-3p. MicroRNAs hsa-miR-10-5p, hsa-miR-146a-5p and has-miR-199b-3 were also the most important hits in the Random Forest analysis

**Fig. 2:**
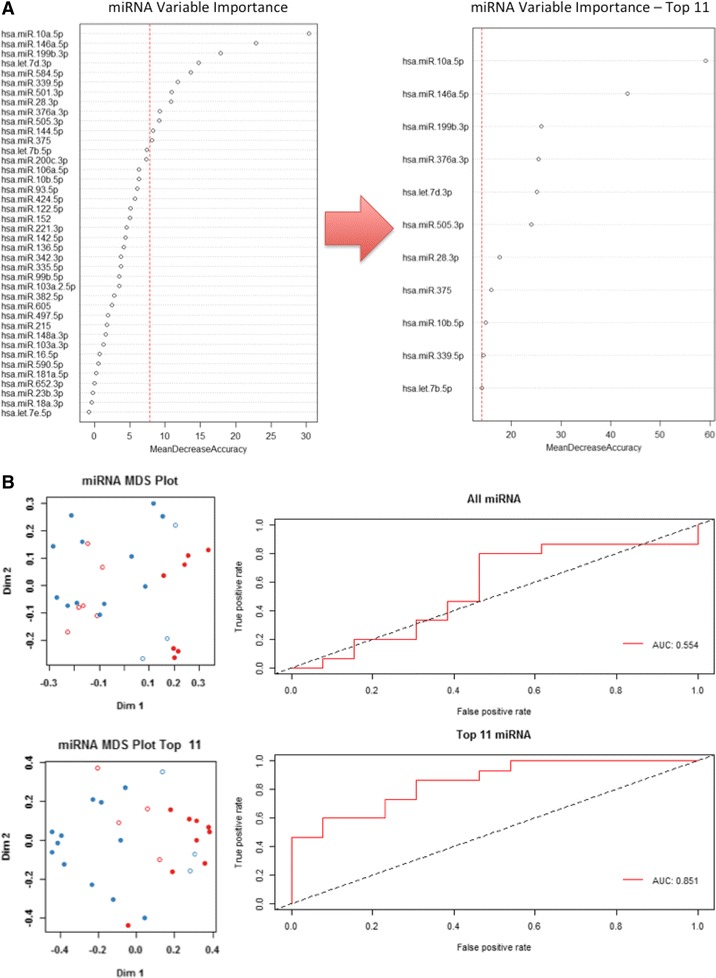
**a** Random Forest analysis classifier of 11 microRNAs. The top three miRNAs in this classifier (hsa-miR-10a-5p, hsa-miR-146a-5p and hsa-miR-199b-3p) were consistent with those identified in the limma and *t*-test statistics. **b** Random Forest Area Under the Curve. AUC using all miRNAs and the 11-miRNA random forest classifier was 0.554 and 0.851, respectively. Red circles represent benign patients, blue represent PTC patients, filled represent correct classification, and open represent incorrect classification

On average, 136 miRNAs were detected per sample and 128 miRNAs qualified for analysis, of which 64 were detected in all 31 samples profiled. After rank invariant normalization followed by Random Forest, t-Test and limma statistical analyses, two miRNAs (hsa-miR-150-5p and hsa-miR-342-3p) were down-regulated and two (hsa-let7b-5p and hsa-miR-191-5p) were up-regulated in benign vs. control samples (Fig. 1a; *p* < 0.001, FDR <5 %). For PTC vs. control samples, one additional microRNA (hsa-miR-146a-5p) was down-regulated and one additional microRNA was up-regulated (hsa-miR-93-5p) (Fig. 1b; *p* < 0.001, FDR <1 %). Importantly, between PTC and benign samples, there was statistically significant down-regulation of hsa-miR-146a-5p and hsa-miR-199b-3p, and up-regulation of hsa-let7b-5p and hsa-miR-10a-5p (Fig. 1c, *p* < 0.05), although the false discovery rate was high (50 %) in this comparison. Random Forest analysis produced a classifier consisting of 11 microRNAs (Fig. 2a), of which miRNAs hsa-miR-10a-5p, hsa-miR-146a-5p and hsa-miR-199b-3p were the most informative. Using this classifier, an Area Under the Curve (AUC) of 0.851 was obtained (Fig. 2b). Results of the limma and t-Test analysis are shown in Table 2.

**Table 2:** Limma statistics and fold change

MiRNA	*p*-value	adj. *p*.Value	*t*-Test	Mean target Ct	Mean calibrator Ct	ddCT	Fold change
Benign vs. Control^a^							
hsa-miR-150-5p	<0.001	<0.001	6.2	30.35	27.79	2.6	−5.9
hsa-miR-342-3p	<0.001	<0.01	4.8	32.34	30.51	1.8	−3.5
hsa-let-7b-5p	<0.001	0.01	−4.0	29.92	31.36	−1.4	2.7
hsa-miR-191-5p	0.001	0.04	−3.5	31.73	32.76	−1.0	2.0
PTC vs. Control^b^							
hsa-let-7b-5p	<0.001	<0.001	−5.6	29.40	31.36	−2.0	3.9
hsa-miR-150-5p	<0.001	<0.001	5.7	30.11	27.79	2.3	−5.0
hsa-miR-146a-5p	<0.001	0.02	3.7	33.49	32.21	1.3	−2.4
hsa-miR-342-3p	<0.001	0.03	3.7	31.88	30.51	1.4	−2.6
hsa-miR-191-5p	<0.001	0.03	−3.5	31.75	32.76	−1.0	2.0
hsa-miR-93-5p	<0.001	0.03	−3.5	28.68	29.53	−0.85	1.8
PTC vs. Benign^c^							
hsa-miR-10a-5p	<0.01	0.5	−3.1	35.45	35.95	−0.50	1.4
hsa-miR-146a-5p	0.01	0.5	2.7	33.49	32.93	0.56	−1.5
hsa-miR-199b-3p	0.01	0.5	2.7	33.33	32.79	0.54	−1.5
hsa-let-7b-5p	0.02	0.5	−2.4	29.40	29.92	−0.51	1.4

Fold-changes and significance of changes by *t*-Test/Limma. ddCT = delta delta CT – measure of relative expression quantification (comparative cycle threshold)

Adjusted *p*-value accounts for multiple comparisons

^a^False discovery rate (FDR) <5 %

^b^FDR <1 %

^c^FDR <50 %

We performed additional statistical testing by adjusting the p-values for multiple comparisons. The miRNA differences between control and benign samples were statistically significant (adjusted *p* < 0.05 for all miRNA), as were the comparison between PTC and control. When this additional statistical analysis was performed, the difference in miRNA between benign and malignant miRNA was no longer significant (*p* > 0.05).

**Fig. 3:**
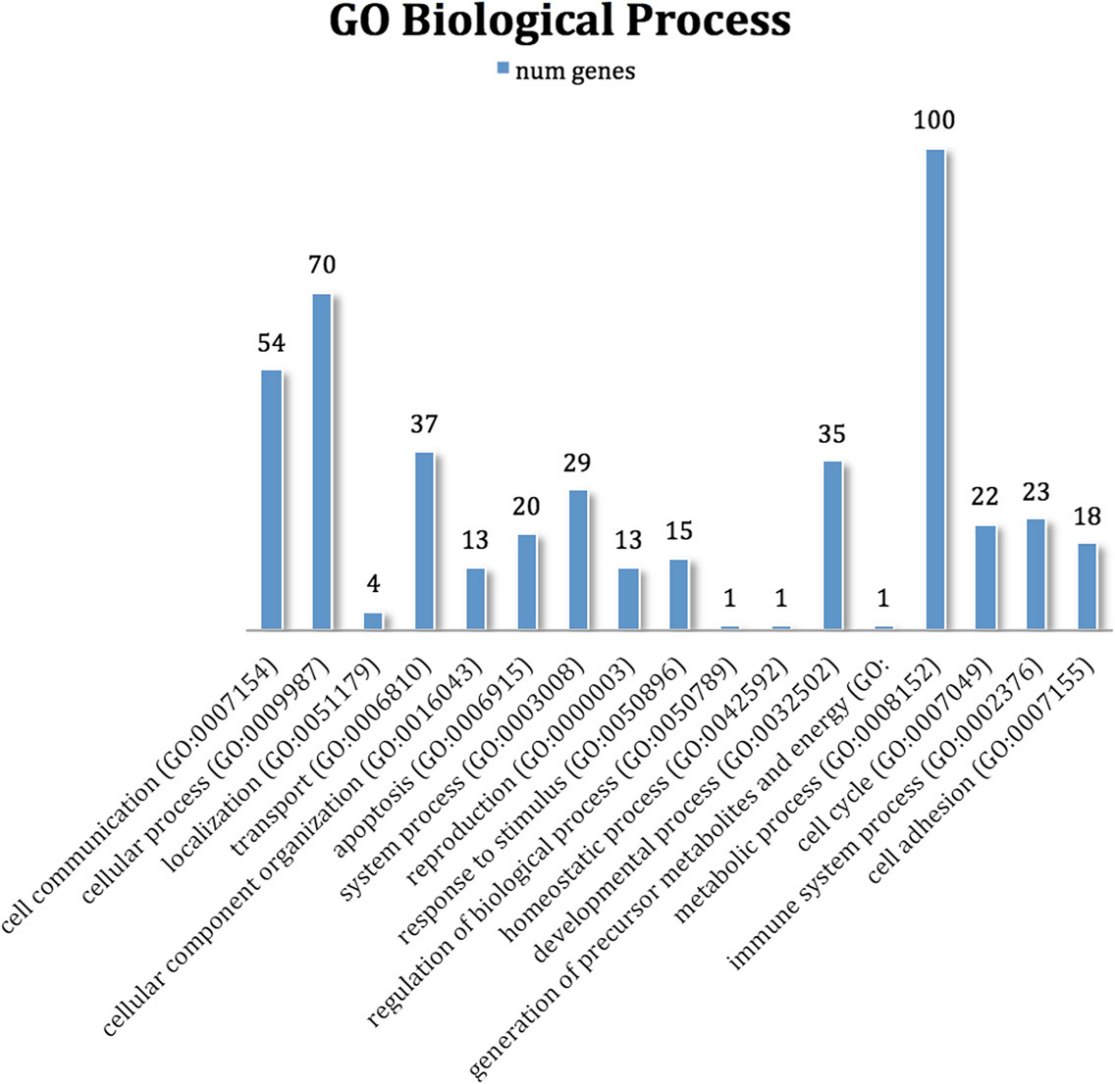
Role of miRNA target genes. Categories of miRNA gene targets from miRDB (microRNA target prediction and annotation database). A prediction score of ≥80 was required

By searching miRDB with the 11 microRNAs in the classifier, 186 unique gene targets were identified. These targets were mostly associated with metabolic process (GO:0008152, 100 targets), cellular process (GO:0009987, 70 targets) and cell communication (GO:0007154, 54 targets) (Fig. 3). Six microRNAs were down-regulated (hsa-miR-146a-5p, −199-3p, −376a-3p, 339-5p, 28-3p, and let-7d-3p), which would result in enhanced expression of the target genes. Five microRNAs were up-regulated (hsa-miR-375, −10b-5p, −10a-5p, −505-3p, and -let7b -5p), which would result in repression of the target genes.

## Discussion

This study identified that the serum of patients with benign thyroid tumors showed statistically significant down-regulation of miR-150-5p and miR-342-3p, and up-regulation of miR-let7b-5p and miR-191-5p compared to controls. The serum of patients with PTC also showed deregulation of these miRNA, with additional down-regulation of miR-146a-5p and up-regulation of miR-93-5p. Most importantly, there was statistically significant down-regulation of miR-146a-5p and miR-199b-3p and up-regulation of let7b-5p and miR-10a-5p when comparing PTC serum to that of patients with benign tumors using limma and t-tests. These are the standard tests used in miRNA literature. Unfortunately, these differences between miRNA between benign and PTC patients did not reach significance with the addition of adjusted p-value for multiple comparators.

The differences in expression levels between the control group and both the benign and PTC groups were the greatest, with average fold changes of 3.5 and 3.0 respectively. However, the average fold change between miRNAs that were differentially regulated between the patients with PTC and those with benign thyroid tumors was only 1.5. The practical impact of this modest difference is a relatively high false discovery rate of 50 %. In other words, two of the four miRNA are likely false positives. Therefore, this panel of miRNAs would require further validation in an independent cohort of patients.

Random Forest plots are used for classification and regression to create predictive models for the variable importance of large numbers of variables. A perfect predictor would have an area under the curve (AUC) of 1, whereas a predictor with an AUC of 0.5 has a predictive value no better than chance. The AUC in the Random Forest plot in PTC versus benign using a set of the 11 most informative miRNA was 0.851. These miRNA target genes were confirmed to be involved in metabolic and cellular processes and cell communication (Fig. 3).

Numerous studies have identified tissue miRNAs involved in PTC tumorigenesis. The majority of these compare PTC tissue to normal thyroid tissue – either from comparative controls or from adjacent “normal” thyroid tissue. He *et al.* were among the first to identify up-regulation of miRNA 146b, 221 and 222 in 20 patients with PTC [28]. miRNA-146b is closely related to miRNA-146a, which was identified in our study. MiR-146a is a known tumor suppressor and its down-regulation appears to enhance tumorigenesis. It plays a role in the classical NF-κB signal transduction pathway in PTC by modulating expression of protein kinase C epsilon (PKCε) and Ras/Raf-1 signaling. It also targets TRAF6 (mirDB score 100) and IRAK1 (mirDB score 87), which form part of a negative feedback loop in NF-κB signaling [29]. Pallate *et al.* also identified the down-regulation of miRNA-181b in PTC tissue, corroborating the results of He’s group [30]. Tetzlaff *et al.* compared PTC tissue to multinodular goiter using formalin fixed paraffin embedded tissue for analysis rather than fresh frozen tissue, and identified 13 up-regulated and 26 down-regulated miRNAs, including those stated above [31].

The let7 family of miRNA was the second group to be identified during the early study of miRNA [32]. Let7b-5p was up-regulated in our study, both in benign and malignant tumors, and exhibited a statistically significant increase when they were compared. It is often significantly up-regulated in various cancers and exerts its effects by enhancing expression of oncogenes such as Ras, c-Myc, cyclin D and HMG [33–35]. Elevated let-7b expression in PTC may have a similar effect on these genes [36–38].

The most significant limitation of our study was its small sample size (13 benign, 18 PTC and 3 controls), limiting our statistical power. This pilot study does have the potential to guide future research into these and other miRNAs to aid in the diagnosis of PTC.

Our study only included the PTC subtype of thyroid carcinoma. Although the diagnostic accuracy for anaplastic and medullary carcinoma is greater on cytopathology alone, follicular carcinoma still remains in the differential diagnosis in non-diagnostic FNA. A limited number of studies have published data on tissue miRNAs in FTC patients. The incidence of FTC is significantly less than that of PTC, therefore obtaining sufficient numbers for meaningful data analysis is more difficult [39, 40].

Although the application of tissue tumor markers such as miRNA and gene profiling may potentially improve diagnostic accuracy, these must be obtained by FNA biopsy and therefore remain invasive, unpleasant and inconvenient for patients. This study suggests miRNA in serum is stable enough for detection, supporting previous evidence to this effect. This would potentially provide clinicians with a novel, non-invasive method to improve diagnostic accuracy while avoiding discomfort to the patients. Serum miRNA for diagnosis would be particularly useful in patients with multinodular goiter. In these patients, there are often numerous thyroid nodules >15 mm and it can be difficult to select which nodules should be biopsied [41]. Serum-based testing would allow thyroid malignancy to be identified much more simply in these patients. A previous study suggests Canadian wait times for thyroid surgery are inappropriately long [42, 43]. Preoperative risk stratification for malignancy will help manage the limited operative time available in our Canadian health care system to expedite care for those more likely to have malignancy.

Serum-based testing in cancer is gaining in popularity as its potential feasibility is recognized. By March 2015, more than 10 papers on this topic had been published in a diversity of tumor types. These include non-small cell lung carcinoma [44, 45], hepatocellular carcinoma [46], ovarian cancer [47], and others. Studies in thyroid cancer have been limited to date. A study by Yu *et al.* in 2012 was the first to examine serum miRNAs in PTC and identified three that were significantly up-regulated in Chinese patients with PTC relative to benign and control patients [48]. This study was limited in that it only aimed to detect 5-fold-changes or more, and only looked for those miRNA that were up-regulated in sera from patients with PTC relative to those with benign disease. A second study in Caucasian patients identified a single miRNA as being significantly up-regulated and one down-regulated in PTC compared to benign sera [49]. These targets, however, were different than the miRNAs identified in the study focusing on Chinese patients.

The previous studies have failed to tightly stratify patients based on clinical characteristics, which can have a significant effect on miRNA expression profiles, although this has not universally been found to be the case [50]. Each study has identified different miRNAs that are differentially expressed in sera of patients with PTC. The populations have all been quite disparate from one another. We have chosen to include exclusively female patients with tumors >1 cm in order to more tightly control differences in baseline miRNA profiles. Nonetheless, the differences in miRNA expression identified between studies suggests a need for a large-scale, tightly controlled study which would serve as a baseline for studies in other populations with different clinical characteristics.

Clinical application of miRNAs may extend to disease monitoring and prognostication. The prognosis of patients with PTC is generally good, with a 10-year survival of 85 % [51]. Some thyroid tumors, such as the tall cell variant, can present aggressively. miRNA detection may identify important molecular information that may determine tumor clinicopathological characteristics and predict aggressiveness of disease and patients at risk of poorer outcomes, and future studies are planned to stratify based on histological subtypes. A previous study has already shown promise in detecting serum miRNAs that may be increased in recurrent PTC [50]. Future miRNA research may even aid in management of PTC patients, given the role of miRNA in cell proliferation, differentiation and apoptosis.

## Conclusion

This study identifies four microRNAs that exhibit statistically significant changes when comparing the serum of patients with PTC to those with benign nodules. These results demonstrate the potential for serum-based miRNA assays to improve diagnostic accuracy of PTC.
